# Department of Therapeutics

**Published:** 1893-06

**Authors:** David Cerna

**Affiliations:** Demonstrator of Physiology in the Medical Department of the University of Texas, etc.


### Abstracts of Current Medical Literature.

### DEPARTMENT OF THERAPEUTICS.

[Under the charge of David Cerna, M. D. Ph. D., Demonstrator of Physiology in the Medical Department of the University of Texas, etc.]

Against Neurastenic Pains.—In the treatment of these conditions, Huchard (*Rev. Internationale de Therapeutique et Pharmacologie*, March 15, 1893,) recommends faradization by means of an electric brush, spraying with chloride of methyl over the vertebral column, and the subcutaneous injections of from 80 to 160 minims (5 to 10 grammes) of an artificial serum prepared according to the following formula:

| | | | |
|---|---|---|---|
| ℞ | Distilled water . . . . | 100.00 | grammes, |
| | Chloride of sodium . . | 6.00 | " |
| | Phosphate of sodium . | 10.00 | " |
| | Sulphate of sodium . . | 2.50 | " |
| | Pure phenic acid . . . | 1.50 | " |

Should the anæmia be pronounced, the author adds to the above treatment a daily injection of 16 minims (1 gramme) of a solution of hæmoglobin of the strength of 1-10 per cent.

---

Sozoiodol for Whooping-Cough.—Insufflations of the sozoiodolate of sodium have been found of great service by Guttmann (*Therap. Monatssch.* No 1, 1893; *Rev. Internationale de Therapeutique et Pharmacologie*, Feb. 15, 1893) in the treatment of whooping-cough in children. The author tried the drug in thirty children. The quantity insufflated into the nose at each treatment was 4 grains (0.25 gramme). The method tended to diminish the number and the frequency of the attacks in the majority of the cases observed.

---

ANTITOXINE IN THE TREATMENT OF TETANUS.—A very interesting case has been recently reported (March 3, 1893) to the Societé Médicale des Hospitaux by Barth and Mayet (*Rev. Int. de Ther. et Pharm.*, March 15, 1893), successfully treated by injection of antitoxine. A young man, eighteen years of age, electrician by occupation, began to exhibit symptoms of tetanus on the 9th of January. There was no traumatic cause. He was admitted to the hospital on the 14th of the same month, in a very serious condition. On the 16th he received at 5 p. m. the first hypodermic injection of 800 minims (50 cubic centimeters) of an antitoxic serum of the power of 10 millions, furnished by Houx of the Pasteur Laboratory. At 8 p. m. a similar injection was administered, and at 10 p. m. a third injection of the same quantity was given. A fourth injection was made on the 17th of January at 8 a. m., and the fifth injection at 5 p. m. On the 19th the patient received the sixth injection at 1 p. m., and on the 21st the seventh and last injection of 480 minims (30 cubic centimeters) of antitoxine. The patient recovered completely by the 10th of February. No apparent local reaction followed the injections. The general reaction consisted in a considerable elevation of the temperature which soon afterwards declined. The definite amelioration was manifested after marked sweating, followed by the appearance of a general urticaria-like eruption which lasted thirty-six hours.

---

THE ANTISEPTIC TREATMENT OF APHTHOUS STOMATITIS.—Bearing in mind that the pathological cause of aphthous stomatitis is identical with the microbe found in aphthous fever of the lower animals, the salicylated treatment for the above affection has been recommended as follows:

1. Touch the affected parts with this:

| | | |
|---|---|---|
| a℞ | Salicylic acid . . . . . | 2.00 grammes, |
| | Glycerine . . . . . . . | 30.00 " |
| | Alcohol, q. s. | |

or with this other combination:

| | | |
|---|---|---|
| b℞ | Sulphoricinate of sodium . | 80.00 grammes, |
| | Salol . . . . . . . . . . . | 20.00 " |

2. During the period of ulceration touch the affected parts with the following:

| | | |
|---|---|---|
| c℞ | Salicylate of sodium . . . | 1.00 gramme, |
| | Hydrochlorate of cocaine . | 1.00 " |
| | Boiling water . . . . . . . | 100.00 " |

3. Administer internally this combination:

| | | |
|---|---|---|
| d℞ | Salicylate of bismuth . . | 1.00 gramme, |
| | Benzo-naphthol . . . . . | 1.20 grammes. |

Mix and make four cachets.

Sig.—One cachet four times a day.

(*Rev. Gener. de Clin. et Therap.; Bull. Genet. de Therapeutique*, March 15, 1893.)

---

DUBOISIN IN PARALYSIS AGITANS.—E. Mendel (*Neurolg. Centralblatt*; *Internat. Medical Magaz.*, May 1893) recommends duboisin in paralysis agitans, not as a remedy, but as a great palliative. After injection of two or three decimilligrammes of the drug the tremor ceases for a period of from three to five hours. Sleep, too, is much improved by it and muscular rigidity decreased. The remedy can be used safely for a long time, and there is no danger of the formation of a drug habit.

---

GUAIACOL IN PHTHISIS.—D. M. Reese (*Internat. Med. Mag.; University Med. Mag.*, May, 1893) reports his experience with guaiacol in phthisis. He employed the drug in sixty-six cases in the out-patient department and in thirty-five in the wards of the Johns-Hopkins hospital. Of the dispensary patients, thirty-five had extensive diseases, and thirty-one had slight involment of one or other apex; thirteen with advanced phthisis, showing some signs of improvement, while in the remaining twenty-two there was no change. Of the thirty-one incipient cases, ten showed distinct improvement. Among the ward patients, thirty-five in number, twenty-one were distinctly benefited. In three cases the drug was given hypodermically in ten minim doses daily, with the view of lowering temperature, but in no instance was it lowered more than one degree an hour after the injection, and the fall was only transient. Pains were experienced at the seat of injection, but in no case was there abscess formation. The only good effects seem to have been temporary reduction of cough and expectoration. In a few cases the physical signs improved, but there did not seem to be any evidence of specific action on the bacillus. In early cases the general nutrition was benefited and the appetite improved. Guaiacol had the advantage over creasote in not causing gastric disturbance.

---

FOR DIARRHŒA.—According to *L' Union Medicale*, Mencke employs the following prescription:

℞ Powdered resorcin . . . . . . . . . grs. xv,
Paregoric . . . . . . . . . . . . . mins. xv,
Distilled water . . . . . . . . . . ℥ iij,
Syrup . . . . . . . . . . . . . . . ℥ ij.

A dessertspoonful of this may be taken every two hours.

In the case of children it is well to diminish the quantity of resorcin and of the paregoric, or a coffeespoonful of this mixture may be given every two hours.—*Therapeutic Gazette*, May 1893.

